# Combustion Synthesis of Magnesium-Aluminum Oxynitride MgAlON with Tunable Composition

**DOI:** 10.3390/ma16103648

**Published:** 2023-05-10

**Authors:** Tigran Akopdzhanyan, Danil Abzalov, Dmitry Moskovskikh, Mohammad Abedi, Valentin Romanovski

**Affiliations:** 1 Macrokinetics of SHS Processes in Reactors, Institute of Structural Macrokinetics and Materials Science ISMAN, Chernogolovka 142432, Russia; abzalovdanil99@yandex.ru; 2 Center of Functional Nano-Ceramics, National University of Science and Technology MISIS, Moscow 119049, Russia; abedi@edu.misis.ru (M.A.); vramano@kth.se (V.R.)

**Keywords:** magnesium-aluminum oxynitride, MgAlON, combustion synthesis, nitrogen atmosphere, transparent ceramics, reaction mechanism

## Abstract

Magnesium-aluminum oxynitride MgAlON has garnered significant attention in recent years due to its unique properties and potential applications. Herein, we report a systematic study on the synthesis of MgAlON with tunable composition by employing the combustion method. The Al/Al_2_O_3_/MgO mixture was combusted in nitrogen gas, and the effects of Al nitriding and oxidation by Mg(ClO_4_)_2_ on the exothermicity of the mixture, combustion kinetics, and phase composition of combustion products were investigated. Our results demonstrate that the MgAlON lattice parameter can be controlled by varying the AlON/MgAl_2_O_4_ ratio in the mixture, which corresponds to the MgO content in the combustion products. This work provides a new pathway for tailoring the properties of MgAlON, which may have significant implications in various technological applications. In particular, we reveal the dependence of the MgAlON lattice parameter on the AlON/MgAl_2_O_4_ ratio. The limitation of the combustion temperature by 1650 °C resulted in obtaining submicron powders with a specific surface area of about 3.8 m/g^2^.

## 1. Introduction

Transparent ceramic materials have attracted significant attention due to their transparency in the ultraviolet (UV), visible (VIS), and infrared (IR) regions of the electromagnetic spectrum, coupled with excellent thermomechanical properties, making them an attractive alternative to traditional materials such as quartz, tempered glass, and sapphire [1]. Moreover, these materials hold great promise for high-temperature applications in the aerospace engineering and power industry [2]. Magnesium aluminum spinel MgAl_2_O_4_ and aluminum oxynitride (AlON) are well-known examples of optically transparent materials [1,3,4,5,6] that, due to their high crack resistance, can be used as transparent armor [7].

Of particular interest in recent years is the relatively new material magnesium-aluminum oxynitride (MgAlON) [8,9,10,11], which lies within the AlN-Al_2_O_3_-MgO ternary phase diagram and is an infinite solid solution of spinel and AlON. The synthesis of MgAlON is typically accomplished using the same methods employed for AlON powder synthesis. The main methods for obtaining MgAlON include solid-state reaction [8,9,11,12,13,14,15,16] and the carbothermal reduction of Al_2_O_3_ in a nitrogen atmosphere [17,18,19,20]. In the solid-state reaction method, mixtures of AlN, Al_2_O_3_, and MgO powders are placed in a high-temperature furnace, and synthesis is carried out at temperatures above 1700 °C for several hours. However, the high cost of the initial components, particularly AlN, is a significant disadvantage of this method. In contrast, the carbothermal reduction method involves using a mixture of carbon, Al_2_O_3_, and MgO powders in the required proportions [17,18,19,20]. The synthesis proceeds at temperatures above 1700 °C for several hours in a nitrogen atmosphere. However, a common disadvantage of both methods is the need for prolonged high-temperature heating, which leads to high energy consumption.

In previous works, MgAlON powder was synthesized using magnesium aluminum silicate as a source of magnesium [13], and the method of aluminothermic reduction and nitriding was used to synthesize MgAlON powder using an Al-Al_2_O_3_-MgO mixture [10]. However, there are no reports on MgAlON combustion synthesis (CS) or self-propagating high-temperature synthesis (SHS) in open sources, which is a highly efficient method of ceramic production, especially for nitride ceramics [21].

In this work, we propose a self-propagating high-temperature synthesis method for the synthesis of MgAlON, similar to the modified method for the synthesis of aluminum oxynitride presented in [22]. This method enables the synthesis of single-phase MgAlON powders through combustion without the need for external high-temperature heating. The only electrical input is required to initiate the combustion process by heating a small nichrome spiral. Once initiated, the combustion synthesis proceeds in a self-propagating mode and lasts no more than 120 s, whereas high-temperature furnaces require at least 30 min for heating, followed by an exposure time of at least 2 h and subsequent cooling, which also takes several hours. The combustion method itself is widely known and utilized globally. Although MgAlON is a transparent ceramic material, the primary goal of this research was to obtain single-phase MgAlON powders. Typically, the production of optically transparent ceramics occurs in two stages: powder preparation and sintering.

In particular, we studied the combustion of Al/Al_2_O_3_/MgO mixtures in a nitrogen atmosphere (5–60 MPa) and optimized the synthesis conditions to obtain a single-phase fine MgAlON powder. Due to the insufficient exothermicity of the reactive mixture, chemical activation [23,24,25,26,27,28,29] was used. It was first proposed by A.G. Merzhanov [23,25]. Additionally, thermally coupled reactions yet chemically separated combustion processes (“chemical oven”) could be used [28,29]. To chemically activate the combustion process and further increase the reaction exothermicity, we added extra Al and Mg(ClO_4_)_2_ into the stoichiometric Al/Al_2_O_3_ mixture. Mg(ClO_4_)_2_ was used as an Al oxidizer but also played the role of the gas-phase transport of reagents in the combustion front [30]. The novelty of the proposed method is in the chemical activation of the combustion wave using highly exothermic additives and the simultaneous oxidation and nitriding of Al. We study the effect of the Mg(ClO_4_)_2_ content, nitrogen pressure, and initial mixture composition on the exothermicity of the mixture, combustion wave velocity, and combustion product composition.

## 2. Materials and Methods

**Figure 1:**
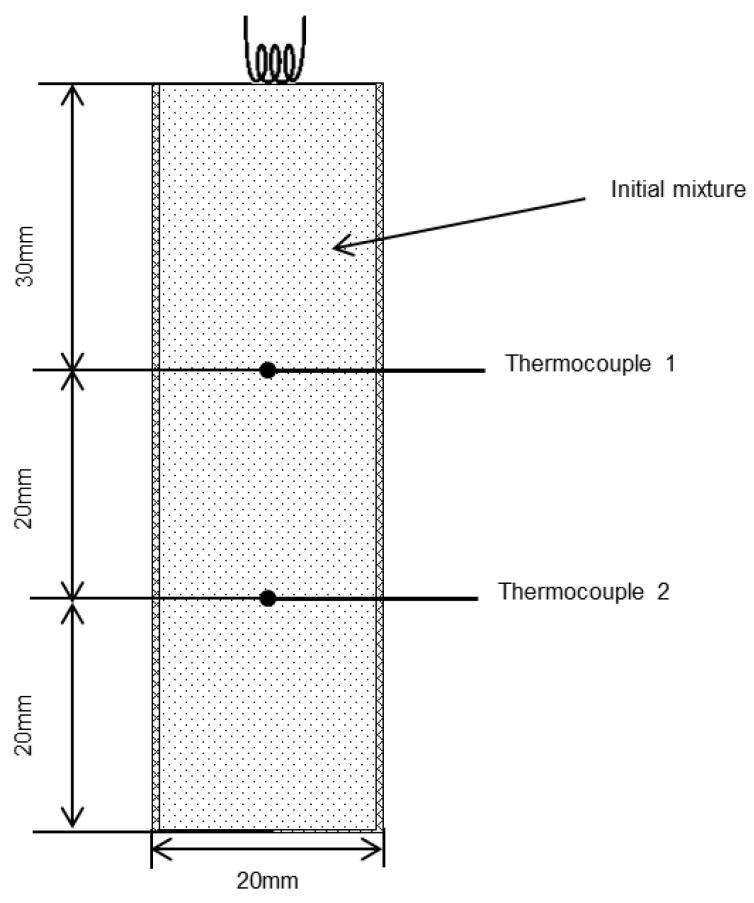
The schematics of the combustion experiments.

In this study, we report the synthesis of AlON/MgAl_2_O_4_ (spinel) solid solutions using commercial powders of Al (purity of 99%, particles size of 5 µm), Al_2_O_3_ (purity of 99.9%, particles size of 55 µm), MgO (purity of 99%, particles size of 5 µm) and Mg(ClO_4_)_2_ (99%) as initial precursors. The powders were carefully weighed and mixed in proportions ranging from 90/10 to 10/90 for the formation of the desired solid solution. To achieve homogeneity, the mixtures were ball-milled for 4 h, followed by calculation to obtain AlON with 35.7 mol. % AlN/64.3 mol. % Al_2_O_3_ and MgAl_2_O_4_ with 50 mol. %Al_2_O_3_/50 mol. %MgO. The reactive mixtures (25 g) were then compacted to a bulk density of 1.1–1.2 g/cm^3^ and placed in a paper cup (d = 20 mm, L = 70 mm). The combustion temperature and velocity were recorded using two W–Re thermocouples, as shown in Figure 1. The location of thermocouples at a distance of 20 mm from each other allows for reducing the measurement error. Additionally, the location of the upper thermocouple at a distance of 30 mm from the top of the sample allows for leveling the influence of the ignition coil on the combustion temperature in this area. Thus, it allows for increasing the accuracy of the temperature and combustion rate measurements by reducing the error. The maximum measurement error was no more than 5% for the combustion temperature and no more than 10% for the combustion rate.

Following the preparation of the reactive mixtures as described above, the samples, which had been fitted with W-Re thermocouples, were placed in a combustion reactor, where it is possible to carry out synthesis processes at initial gas pressures up to 200 MPa. The reactor was first evacuated and then filled with high-purity nitrogen (>99.9%) at pressures ranging from 5 to 60 MPa. To ignite the reactive mixtures, an 80%Ni-20%Cr wire coil was employed. The combustion and nitriding of the mixtures occurred rapidly, with complete reaction times ranging from approximately 15 to 120 s. Upon cooling, the products were crushed and sifted using a 120 μm sieve.

The combustion products were then subjected to a range of analytical techniques to characterize their structure and properties. Specifically, X-ray phase analysis Dron-3M (Burevestnik, St. Petersburg, Russia), chemical analysis, a scanning electron microscopy SEM LEO-1450 (Carl Zeiss, Oberkochen, Germany) scanning electron microscope with the built-in INCA ENERGY 350 EDS analyzer (Oxford Instruments, Abingdon, UK), and a Rietveld refinement were employed to investigate the composition and microstructure of the combustion products. The lattice parameters of analyzed phases were calculated using the Rietveld refinement of XRD patterns. Additionally, the specific surface area of the combustion products was determined using the nitrogen three-point BET Nova 1200 (Quantachrome Instruments, Boynton Beach, FL, USA). The particle size distribution of the obtained powders was measured using a laser particle size analyzer Fritsch Analysette 22 MicroTec plus (FRITSCH GmbH, Idar-Oberstein, Germany). Overall, this comprehensive suite of analytical techniques provided detailed insights into the properties and structure of the combustion products and allowed us to better understand the mechanism of the combustion process.

## 3. Results and Discussion

### 3.1. Combustion Synthesis of MgAlON Powders: Macrokinetic Parameters and Reaction Mechanisms

In the pursuit of obtaining high-quality MgAlON powders, we employed a novel approach that involves synthesizing the AlON/spinel solid solution. Specifically, we prepared mixtures of Al, Al_2_O_3_, MgO, and Mg(ClO_4_)_2_ in varying proportions, followed by the initiation of two exothermic reactions, namely, nitriding and aluminum oxidation. By controlling the ratio between AlON and Spinel, we could obtain MgAlON powders of various compositions.

However, the decrease in the AlON content leads to a reduction in the amount of aluminum participating in the nitriding reaction, which significantly affects the combustion temperature and rate. Moreover, the temperature increase results in grain growth, which can have a detrimental effect on the properties of the final ceramics, particularly the optical properties.

Therefore, our primary objective was to investigate the individual effects of oxidation and nitriding reactions on the combustion temperature to gain precise control over the process. This would allow us to obtain single-phase MgAlON powders of any composition, ranging from pure AlON to pure MgAl_2_O_4_ spinel, while maintaining the same grain size. We designated the resulting mixtures as Mga10, Mga20, Mga30, etc., where the number signifies the calculated weight percent of the spinel in the AlON/spinel solid solution.

**Figure 2:**
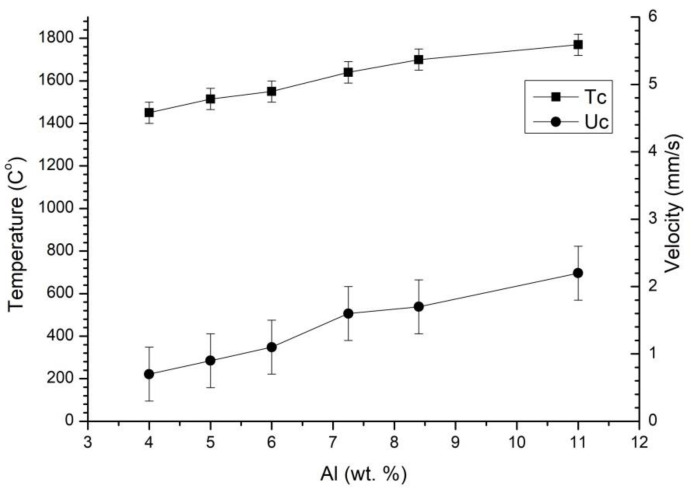
The dependence of the combustion temperature Tc and combustion front propagation velocity Uc on the aluminum content (wt.%) involved in the nitriding reaction.

Figure 2 shows the influence of the aluminum content involved in the nitriding reaction on the dependence of the combustion temperature and combustion front propagation velocity.

Table 1 displays the compositions of the mixtures used in Figure 2. It is essential to emphasize that the amounts of aluminum, which participates in the oxidation reaction, and magnesium perchlorate were kept constant in the mixtures. This approach enables the fixation of the energy released due to the oxidation reaction, allowing for a better understanding of the thermal effect associated with the nitriding reaction. The experiments presented in this study were conducted under a nitrogen pressure of 5 MPa.

The graph in Figure 2 shows that with a 1 wt.% increase in aluminum involved in the nitriding reaction, the combustion temperature and combustion rate increase by 50–70 °C and ~0.5 mm/s, respectively. This information can be used to estimate how the combustion rate and temperature will change with an increase in the proportion of the spinel in the AlON/MgAl_2_O_4_ ratio during the MgAlON synthesis. The combustion temperature and combustion front propagation velocity changed linearly, but as noted in [22], the combustion temperature in this system is limited by the melting temperature of aluminum oxide.

However, if the mass of aluminum involved in the nitriding reaction decreases below 7 wt.% (which corresponds to the composition of Mga40 or the weight ratio of 3/2 AlON/MgAl_2_O_4_), the combustion temperature decreases below the required 1700 °C. This can lead to the incomplete formation of the solid solution and the formation of a multiphase powder in the combustion products. To increase the combustion temperature and completeness of the MgAlON formation, the proportion of aluminum and magnesium perchlorate can be increased while simultaneously reducing the amount of aluminum oxide to maintain the required ratio of components.

**Figure 3:**
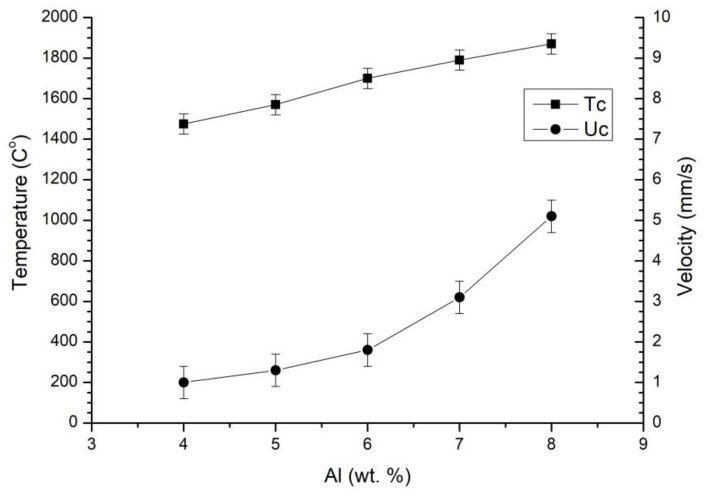
The dependence of the combustion temperature Tc and combustion front propagation velocity Uc on the aluminum content (wt.%) involved in the oxidation reaction.

Figure 3 shows the dependence of the combustion temperature Tc and combustion front propagation velocity Uc on the aluminum content (wt.%) involved in the oxidation reaction by magnesium perchlorate. To investigate the impact of the oxidation reaction, mixtures with a fixed amount of aluminum involved in the nitriding reaction and varying amounts of magnesium perchlorate and aluminum involved in the oxidation reaction were utilized. By maintaining a fixed amount of aluminum in the nitriding reaction, only one target phase is formed. However, this approach allows us to fix the energy released due to the nitriding reaction and assess the thermal effect of the oxidation reaction. Table 2 displays the compositions of the mixtures used in Figure 3.

**Figure 4:**
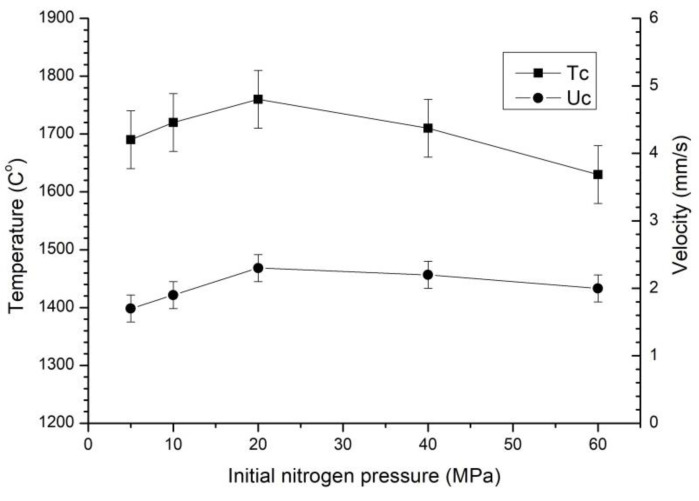
Dependence of the combustion temperature and velocity on the starting nitrogen pressure.

An increase in the wt.% of the aluminum involved in the oxidation reaction causes the combustion temperature to increase by 100–150 °C per 1 wt.% of aluminum. This indicates that the oxidation reaction has a greater effect on the combustion temperature than the nitriding reaction. The combustion velocity does not change linearly due to an increase in the number of magnesium perchlorate decomposition products in the combustion zone, which leads to an increase in gas pressure in the combustion front. The role of magnesium perchlorate and the mechanism of its reaction with aluminum are discussed in [22]. The initial pressure of the reacting gas can significantly impact the macrokinetic parameters. Figure 4 shows the relationship between the combustion temperature Tc and the combustion front propagation velocity Uc and the starting nitrogen pressure for mixture Mga30. The results indicate that the initial pressure of the reacting gas plays a crucial role in the reaction dynamics. The mixture Mga30 contained 14.4 wt.% Al, 68.4 wt.% Al_2_O_3_, 8.2 wt.% MgO, and 9.0 wt.% Mg(ClO_4_)_2_, with 8.4 wt.% of Al used in the nitriding reaction and 6 wt.% oxidized by Mg(ClO_4_)_2_.

The observed difference in the combustion temperature Tc and combustion front propagation velocity Uc can be attributed to the hindered filtration of nitrogen into the combustion front. Interestingly, both Uc and Tc showed an increase of up to 20 MPa but then decreased with a further increase in the initial nitrogen pressure. This fact can be explained by the influence of the high pressure of nitrogen. It hinders the release of gaseous admixtures from the combustion front, which inhibits the spread of the fusible components (Al for example) melt in the front and reduces the combustion front propagation velocity. Moreover, 20 MPa of initial nitrogen pressure overcomes the filtration difficulties and prevents nitrogen shortage during combustion, leading to an increase in Tc (up to 1760 °C) and Uc.

These findings suggest that a pressure of 20 MPa is enough for the nitriding process of these mixtures, and further increasing the nitrogen pressure is not necessary. However, higher nitrogen pressures lead to increased heat losses from the combustion front due to the higher heat conductivity of the atmosphere, which ultimately results in diminishing combustion velocities Uc.

### 3.2. MgAlON Powders: Phase Composition of Combustion Products

Utilizing the X-ray diffraction (XRD) analysis of combustion products can be useful in establishing the phase composition and combustion conditions, including temperature and velocity.

**Figure 5:**
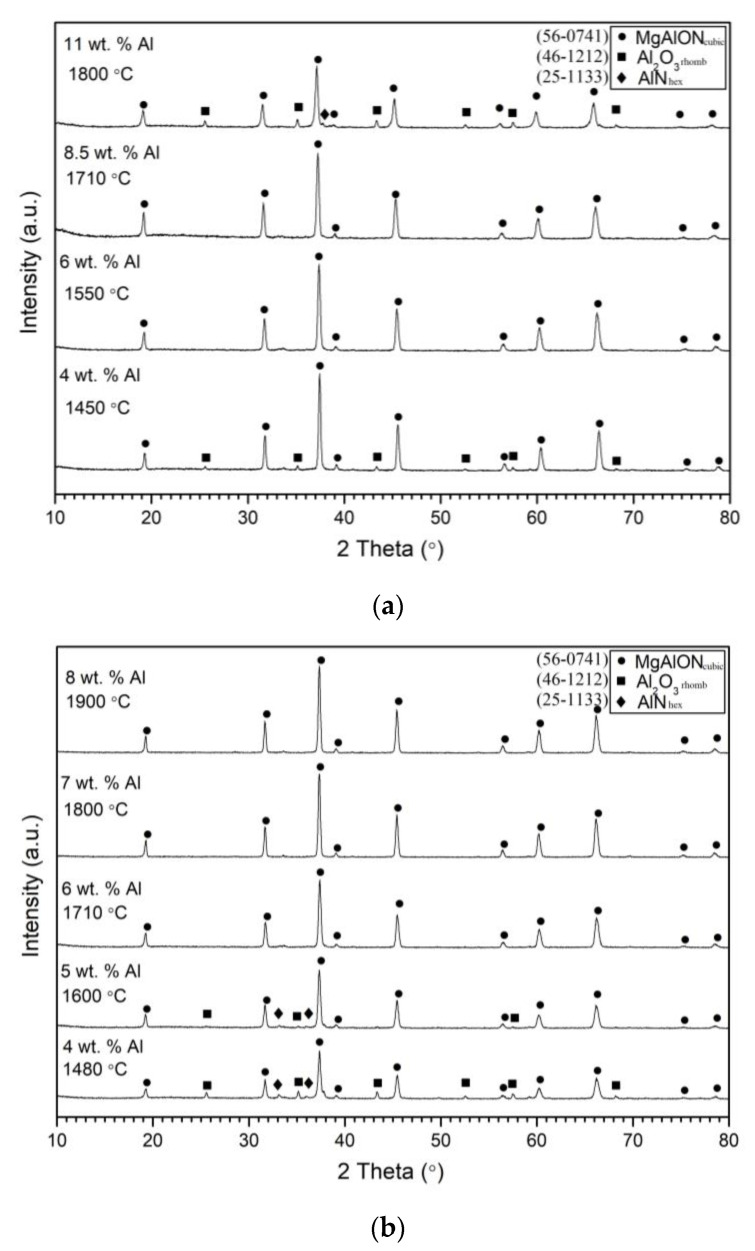
XRD diagrams of combustion products obtained from a mixture with different amounts of Al involved in the nitriding reaction (**a**) and oxidation reaction (**b**).

Figure 5a displays the phase composition of combustion products obtained at a 5 MPa nitrogen pressure from mixtures containing various amounts of Al involved in the nitriding reaction, along with the corresponding combustion temperatures. The figure reveals that a single-phase MgAlON powder was obtained above ~1550 °C. In contrast, combustion products obtained with different amounts of Al involved in the oxidation reaction by Mg(ClO_4_)_2_ (Figure 5b) showed a different pattern: single-phase material was obtained during combustion at temperatures above ~1700 °C. XRD analysis also confirmed the lack of reactivity in the temperature range below ~1550 °C, revealing the presence of Al_2_O_3_ peaks and no presence of Al peaks, indicating the completion of the nitridation reaction at P_N2_ = 5 MPa. It is worth noting that in a previous study [22], peaks of unreacted Al were detected in the combustion product of AlON due to the use of a mixture with a higher amount of Al, leading to aluminum melting and the appearance of filtration difficulties.

**Figure 6:**
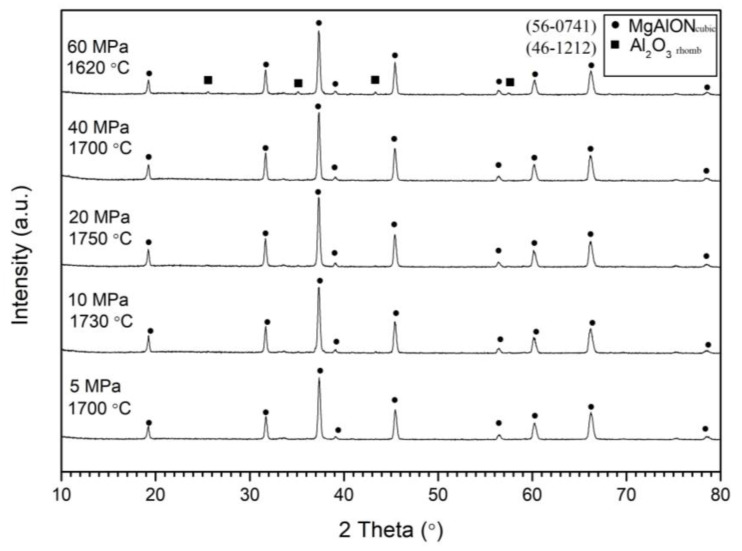
XRD of combustion products obtained from the Mga30 mixture (Table 1) at different initial nitrogen pressures.

As previously demonstrated, the pressure of the reacting gas played a crucial role in the combustion parameters. Figure 6 presents the XRD diagram of combustion products obtained at different nitrogen pressures ranging from 5 to 60 MPa. The results indicated that a single-phase powder was obtained at pressures of 40 MPa and below. However, at a pressure of 60 MPa, traces of unreacted alumina were detected, despite the combustion temperature exceeding the previously mentioned ~1550 °C. This can be attributed to the elevated pressure causing a high rate of heat loss, impeding the completion of the reaction.

### 3.3. Combustion Synthesis of Single-Phase MgAlON Powders

Using the obtained data shown above, we revealed the effect of the composition of the initial mixtures on the temperature and combustion rate and, as a consequence, on the phase composition. To obtain MgAlON of various compositions from Mga10 to Mga90, we need to change the amount of aluminum nitride, as well as as a result of the aluminum involved in the nitriding reaction. We can control the combustion temperature by changing the amount of aluminum involved in the oxidation reaction and the oxidizer. It was found that approximately 1 wt.% of aluminum involved in the oxidation reaction changes the combustion temperature by ~100–120 °C; using the available data, we calculated the initial mixtures. The mixture compositions are presented at Table 3. The experiments were carried out at an initial gas pressure of 5 MPa.

**Figure 7:**
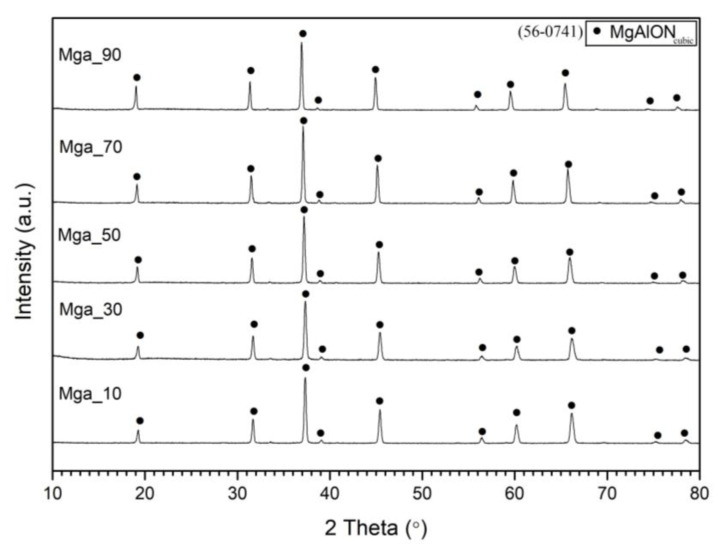
XRD diagrams of the combustion products of MgAlON with different AlON:MgAlON weight ratios.

Figure 7 shows the XRD diagrams of the combustion products of these mixtures. As follows from these graphs, we obtained single-phase MgAlON of various compositions.

**Figure 8:**
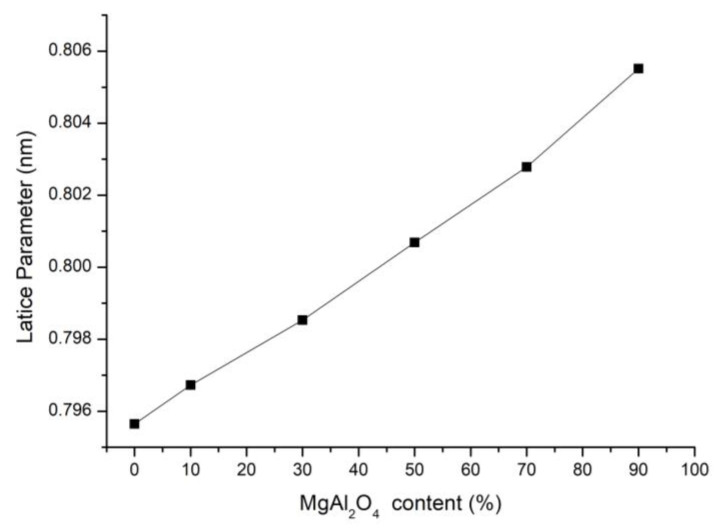
The dependence of the MgAlON lattice parameter on the MgAl_2_O_4_ content.

The combustion products, despite having similar combustion temperatures, showed differences in the lattice constants of the MgAlON phase, which are attributed to the MgAlON composition. The increase in the MgAl_2_O_4_ content in the AlON/MgAl_2_O_4_ composition led to a gradual increase in the MgAlON lattice parameter, from 0.7956 nm for pure AlON to 0.8055 nm for MgAlON with a 1:9 AlON/MgAl_2_O_4_ ratio, as shown in Figure 8. According to the PDF2 database (2022), the lattice parameter for cubic AlON is between 0.7900 nm and 0.7953 nm, and that for the MgAl_2_O_4_ spinel is between 0.7800 nm and 0.8209 nm.

**Figure 9:**
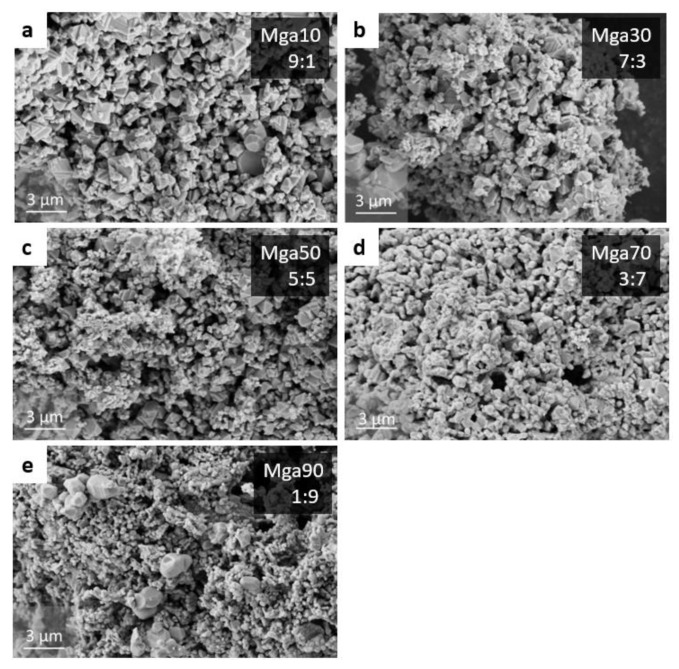
Microstructure of MgAlON powder with 9:1 (Mga10) (**a**), 7:3 (Mga30) (**b**), 5:5 (Mga50) (**c**), 3:7 (Mga70) (**d**), and 1:9 (Mga90) (**e**) ratios of AlON:MgAl_2_O_4_ obtained at P_N2_ = 5 MPa.

Furthermore, Figure 9 presents the microstructure of the obtained single-phase MgAlON powder. By limiting the combustion temperature to 1650 °C, a fine-grain powder with a grain size of <2 μm was obtained, with a specific surface area of 3.8 m/g^2^. This is particularly important for the production of highly transparent optical ceramics. It can be seen from the SEM images that small amounts of MgAl_2_O_4_ in the MgAlON resulted in a sharper structure, while for the Mga70 and Mga90 samples, a noticeable rounding of the grain surface occurs. It should be noted that fine-grain powders are extremely necessary to obtain highly transparent optical ceramics.

Table 4 presents the results of the EDS analysis for the MgAlON powders Mga10, 30, 50, 70, and 90. The compositions correspond to the target compositions of MgAlON powders. As the spinel content in MgAlON increases, the amount of nitrogen decreases, while the magnesium content increases. 

**Figure 10:**
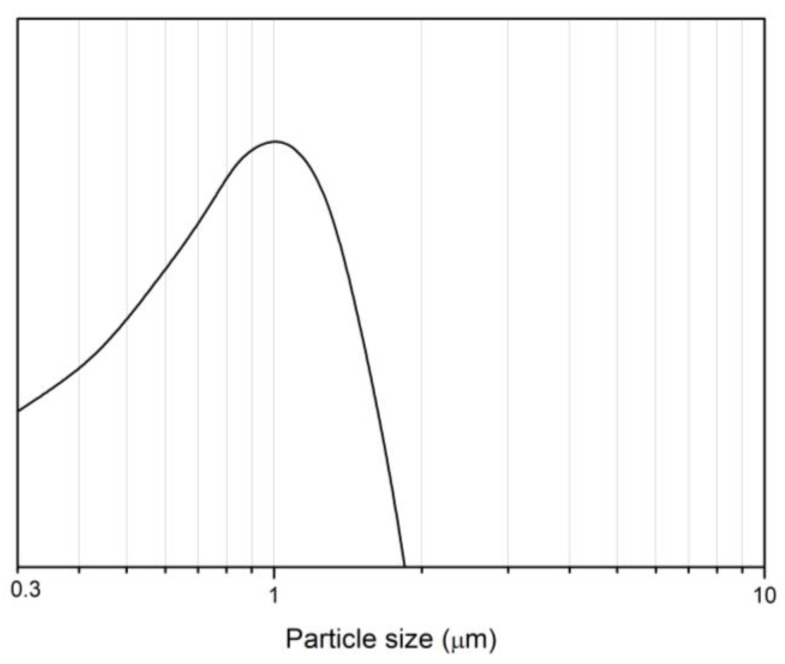
Particle size distribution of the MgAlON powders after ball milling in a planetary mill at 100 rpm for 1 h.

Considering the fine-grained microstructure of the obtained materials, a particle size distribution study was conducted using laser diffraction particle size analyzers, as shown in Figure 10.

To prevent the measurement of large agglomerates, the powders were dispersed in the planetary mill using Si_3_N_4_ jars and balls at 100 rpm for 1 h. The average particle size distribution (d50) was 0.7 µm, and the d90 was less than 1.5 µm. Achieving similar results with powders obtained through carbothermal reduction or solid-state synthesis methods would require long-term grinding (up to 48 h) in a planetary ball mill at 200 rpm using Si3N4 jars and balls, potentially leading to sample contamination with grinding media materials. Moreover, in this case, a significant number of nanosized particles can be observed. These highly reactive particles can activate the sintering process and potentially reduce the sintering time required to obtain fully dense ceramics.

**Figure 11:**
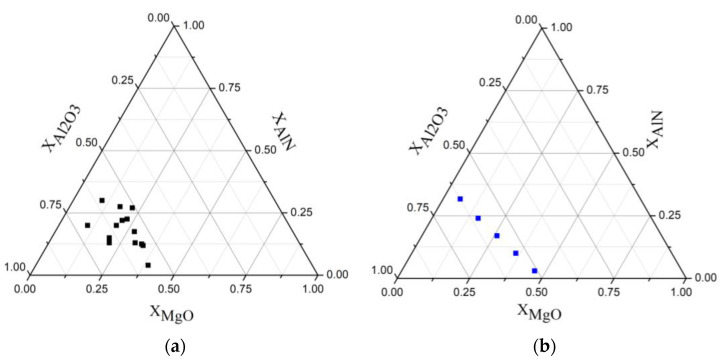
Starting compositions (mole fractions) for the synthesis of MgAlON from selected literature sources [12,16,31,32,33,34] (**a**) and starting compositions (mole fractions) for the synthesis of MgAlON used in this work (**b**).

Lastly, Figure 11 displays the starting compositions (mole fractions) for the synthesis of MgAlON from selected literature sources [12,16,31,32,33,34] (a) and the starting compositions (mole fractions) for the synthesis of MgAlON used in this work (b), shown in a simplified diagram of the ternary MgO-AlN-Al_2_O_3_ system.

## 4. Conclusions

In this study, we have presented a novel method for the production of single-phase MgAlON with various compositions by magnesium perchlorate-activated combustion synthesis. Our approach involved the addition of Mg(ClO_4_)_2_ to over-stoichiometric Al, resulting in an increase in the combustion temperature of the green mixtures up to 1900 °C. Our investigation of the macrokinetics of the combustion process in the Al-Al_2_O_3_-MgO-Mg(ClO_4_)_2_ system revealed that the aluminum nitriding and oxidation reactions played significant roles in the formation of MgAlON. Moreover, we studied the effect of the initial pressure of reacting nitrogen gas on the macrokinetic parameters and phase composition of the combustion products. Our proposed method successfully produced single-phase MgAlON powders with a specific surface area of 3.8 m^2^/g and various compositions. The average particle size distribution of the obtained powders (d50) was 0.7 µm, and the d90 was less than 1.5 µm Furthermore, we established a dependence of the lattice parameter in MgAlON on the spinel content. The lattice parameter increased from 0.79673 nm for MgAlON with 10 wt.% of MgAl_2_O_4_ to 0.80551 nm for MgAlON with 90 wt.% of MgAl_2_O_4_. These results provide valuable insights into the crystal structure and properties of MgAlON, which are essential for its practical applications. Overall, our study presents an innovative method for the synthesis of single-phase MgAlON with various compositions and provides a fundamental understanding of the combustion process and crystal structure of MgAlON. These findings could have significant implications for the development of new materials for various applications, such as ceramic cutting tools.

## Figures and Tables

**Table 1:** The compositions of the mixtures used in graph 2.

Target Phase	Al (Used in Nitriding Reaction), wt.%	Al_2_O_3_, wt.%	MgO, wt.%	Al (Used in Oxidation Reaction), wt.%	Mg(ClO_4_)_2_, wt.%
Mga10	11.1	71.4	2.7	5.8	9
Mga30	8.6	68.4	8.2	5.8	9
Mga40	7.4	66.9	10.9	5.8	9
Mga50	6.2	65.4	13.6	5.8	9
Mga60	4.9	63.9	16.4	5.8	9
Mga70	3.7	62.4	19.1	5.8	9

**Table 2:** The compositions of the mixtures used in graph 3.

Target Phase	Al (Used in Nitriding Reaction), wt.%	Al_2_O_3_, wt.%	MgO, wt.%	Al (Used in Oxidation Reaction), wt.%	Mg(ClO_4_)_2_, wt.%
Mga30	8.6	73	8.2	4	6.2
Mga30	8.6	70.7	8.2	5	7.5
Mga30	8.6	68.2	8.2	6	9
Mga30	8.6	65.8	8.2	7	10.4
Mga30	8.6	63.4	8.2	8	11.8

**Table 3:** The compositions of the mixtures used to obtain single-phase MgAlON of different compositions at P_N2_ = 5 MPa.

Target Phase	Al (Used in Nitriding Reaction), wt.%	Al_2_O_3,_ wt.%	MgO, wt.%	Al (Used in Oxidation Reaction), wt.%	Mg(ClO_4_)_2_, wt.%
Mga10	11.2	74.9	2.7	4.4	6.8
Mga30	8.6	70.7	8.2	5	7.5
Mga50	6.1	64.4	13.6	6.2	9.7
Mga70	3.7	59.2	18.9	7.2	11
Mga90	1.2	54.1	24.1	8.1	12.5

**Table 4:** Results of EDS analysis (wt.%), lattice parameters (nm), and specific surface area (m/g^2^) of obtained MgAlON powders.

Target Phase	N	O	Mg	Al	Lattice Parameter, nm	Specific Surface Area, m/g^2^
MgA10	3.3	44.4	2.1	50.2	0.79673	3.8
MgA30	2.5	43.3	4.9	49.3	0.79853	3.8
MgA50	1.8	44.7	7.9	45.6	0.80069	3.8
MgA70	1.1	43.9	11.1	43.9	0.80278	3.8
MgA90	0.4	46.2	14.4	39	0.80551	3.8

## Data Availability

Data sharing is not applicable.
